# Cervical and Breast Cancer Screening Discussions With Female Adult Inpatients: An Audit Cycle

**DOI:** 10.1192/bjo.2025.10644

**Published:** 2025-06-20

**Authors:** Leanne O’Neil, Vindhya Mampitiya, Akeem Sule

**Affiliations:** 1 Essex Partnership University Trust, Chelmsford, United Kingdom; 2 Lead Employer, East of England, Chelmsford, United Kingdom

## Abstract

**Aims:** To assess whether discussions about breast and cervical cancer screening occur during female patient admissions to acute inpatient wards.

To evaluate the impact of an intervention on the rate of completion of these discussions.

To explore the obstacles which limit these discussions

**Methods:** Female adult patients aged 25–70 years old, admitted to Acute Adult Psychiatric Inpatient Wards (Galleywood, Topaz), Mother and Baby Unit (Rainbow), or Psychiatric Intensive Care Unit (Christopher Unit) between 1/2/2022 and 30/4/2022 were included in baseline data collection (n=57).

Patients aged 25–54 were eligible for cervical cancer screening discussions and aged 50–70 years old for breast cancer screening discussions.

Physical health check proforma and ward review entries were reviewed.

Resident doctors completed a survey to identify barriers to completing these discussions.

An intervention involving enhancing teaching during induction for resident doctors and local academic teaching was implemented over 9 months.

A re-audit was conducted with admissions meeting the same criteria during the period 1/12/2023 to 29/02/2023 (n=34).

**Results:** In the baseline data, 4 out of 43 patients (9%) who were eligible for cervical cancer screening and consented to having a physical health check were included in discussions about cervical cancer screening. This increased to 5 out of 26 (19%) patients during the re-audit.

In the baseline data, 1 out of 15 patients (7%) who were eligible for breast cancer screening and consented to having a physical health check were included in discussions about breast cancer screening. Completion rate remained low with no discussions taking place with the 5 eligible and consenting patients during the re-audit.

Feedback from resident doctors included that there was not enough time to ask these questions during admission, that the patient was unable to answer these questions or that they felt the questions were not relevant.

**Conclusion:** There was a slight increase in cervical cancer screening discussions but a reduced number of breast cancer screening discussions that took place following the intervention. Rates of completion remained low for both.

Clinician feedback highlighted that improvements could be made to understanding the importance of these conversations and alterations to the timing of when they took place.

Further teaching has been implemented, reminder posters have been displayed in all doctors’ offices and options for the timing of completion of these discussions have been recommended.

